# Supplementary material for: Local actin nucleation tunes centrosomal microtubule nucleation during passage through mitosis
Source: EMBO J. 2019 Apr 23;38(11):e99843. doi: 10.15252/embj.201899843 (PMC6545563; doi:10.15252/embj.201899843)
Supplement: Supplementary file 1 — Expanded View Figures PDF [file EMBJ-38-e99843-s001.pdf]

Expanded View Figures

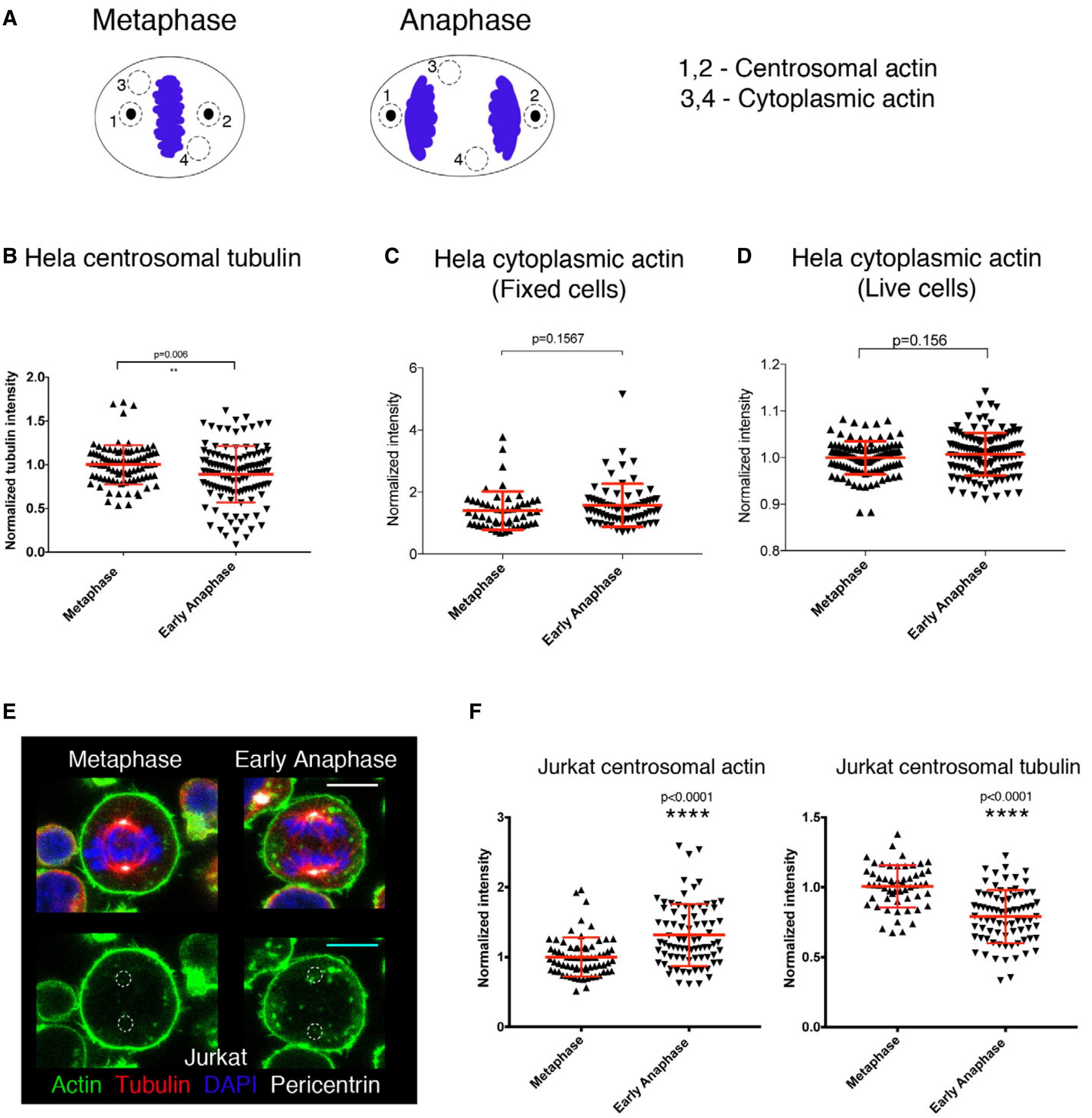

Figure EV1.

© 2019 The Authors

Figure EV1. Actin dynamics during mitotic exit in fixed cells.

A Schematic representation of metaphase and anaphase cell explaining the pools of actin measured in fixed cells. 1 and 2 refer to centrosomal actin, while regions 3 and 4 measure non-centrosomal or cytoplasmic actin.

B Quantification of tubulin intensity around pericentrin-positive centrosomes in HeLa cells immunostained as in (Fig 1A), showing the decrease in tubulin intensity around centrosomes at early anaphase. Mean tubulin metaphase = 1 ± 0.02363, *n* = 90; mean tubulin anaphase = 0.89 ± 0.02937, *n* = 120; Student's *t*-test, *P* = 0.006.

C Quantification of non-centrosomal actin during metaphase and anaphase in fixed HeLa cells (as in Fig 1A and B, Scheme in EV1A) shows that there is no significant increase in the amount of cytoplasmic actin during this period. Mean actin intensity at metaphase = 1.403 ± 0.086, *n* = 52; mean actin intensity at anaphase = 1.574 ± 0.08301, *n* = 70, *P* = 0.1567. All values were normalised to mean actin intensity around centrosomes at metaphase. Error bars indicate standard deviation.

D Quantification of non-centrosomal actin during metaphase and early anaphase in live HeLa cells (as in Fig 1D and E, Scheme EV1A) shows that there is no significant increase in the amount of non-centrosomal actin during this period. Mean actin intensity at metaphase = 0.9994 ± 0.003416, *n* = 108 and mean actin intensity at anaphase = 1.007 ± 0.004158, *n* = 120. *P* = 0.1561, Welch's *t*-test. All values were normalised to mean actin intensity around centrosomes at metaphase. Error bars indicate standard deviation.

E Maximum projection (2 *z*-slices) of Jurkat cells immunostained for F-actin (phalloidin), tubulin, pericentrin and DAPI at metaphase and early anaphase showing the actin accumulation around centrosomes in early anaphase. Scale bar = 10µm

F Quantification of F-actin (phalloidin) and tubulin intensity around pericentrin-positive centrosomes in Jurkat cells immunostained as in (D), showing the increase in F-actin around centrosomes at early anaphase with a decrease in tubulin. Mean actin metaphase = 1 ± 0.03351, *n* = 70; mean actin anaphase = 1.315 ± 0.04993, *n* = 79; mean tubulin metaphase = 1.006 ± 0.02017, *n* = 56; mean tubulin anaphase = 0.7905 ± 0.02144, *n* = 78, Student's *t*-test for both, ****$P < 0.0001$.

© 2019 The Authors

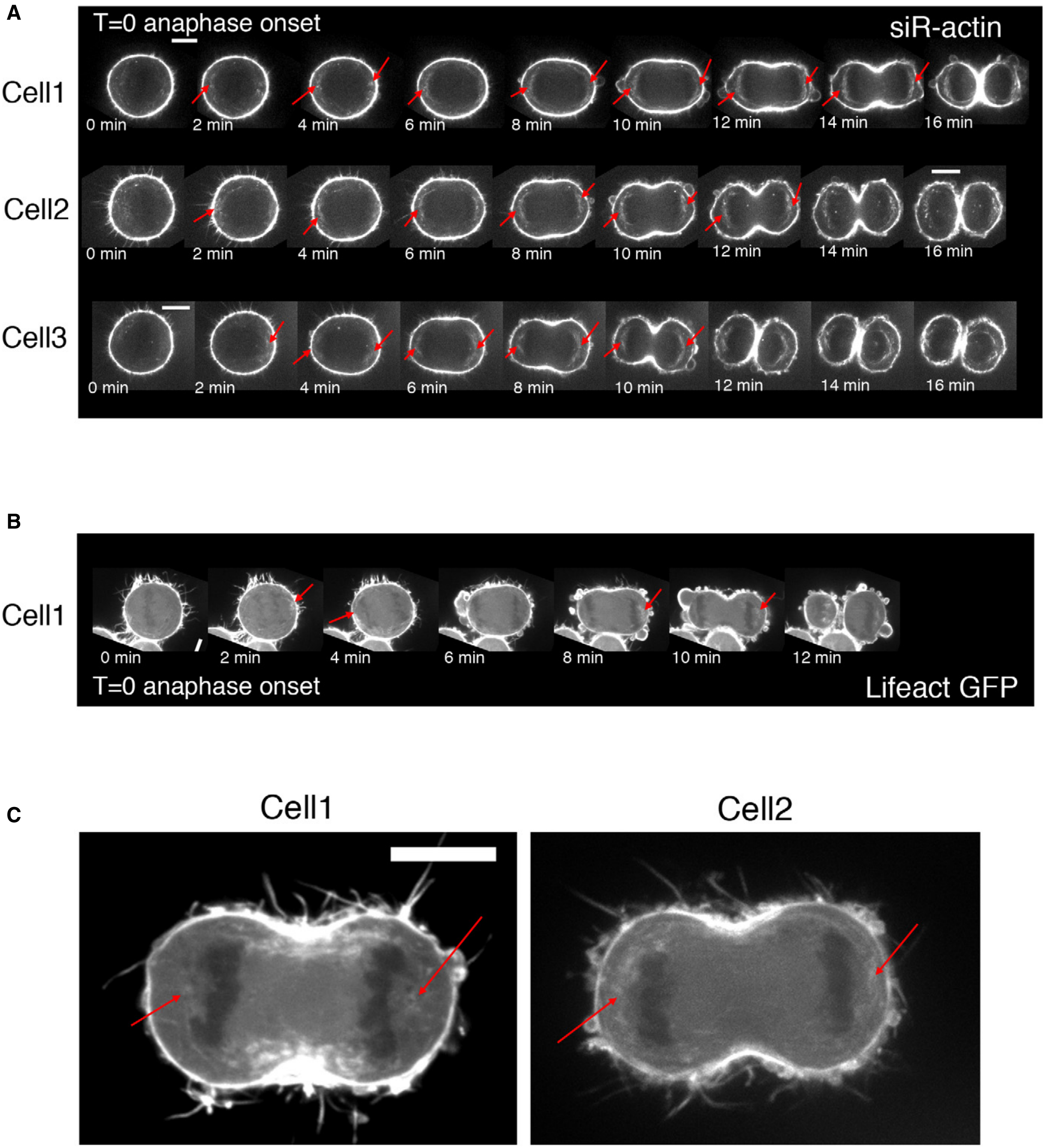

Figure EV2. Actin dynamics during mitotic exit in live cells.

A Representative images of time lapse of HeLa cells imaged with siR-actin, every 1 min. Red arrows point to actin in the presumptive centrosomal region. Note that with siR-actin, this pool of actin around centrosomes persists for a longer time.

B Representative images of time lapse of HeLa cells expressing Lifeact-GFP, imaged every 1 min. Red arrows point to presumptive centrosomal region. This reporter shows a more widespread accumulation of cytoplasmic actin, with frequent enrichment around one or both centrosomes.

C Stills from time lapse of HeLa cells expressing Lifeact-GFP, showing actin enrichment around both centrosomes.

© 2019 The Authors

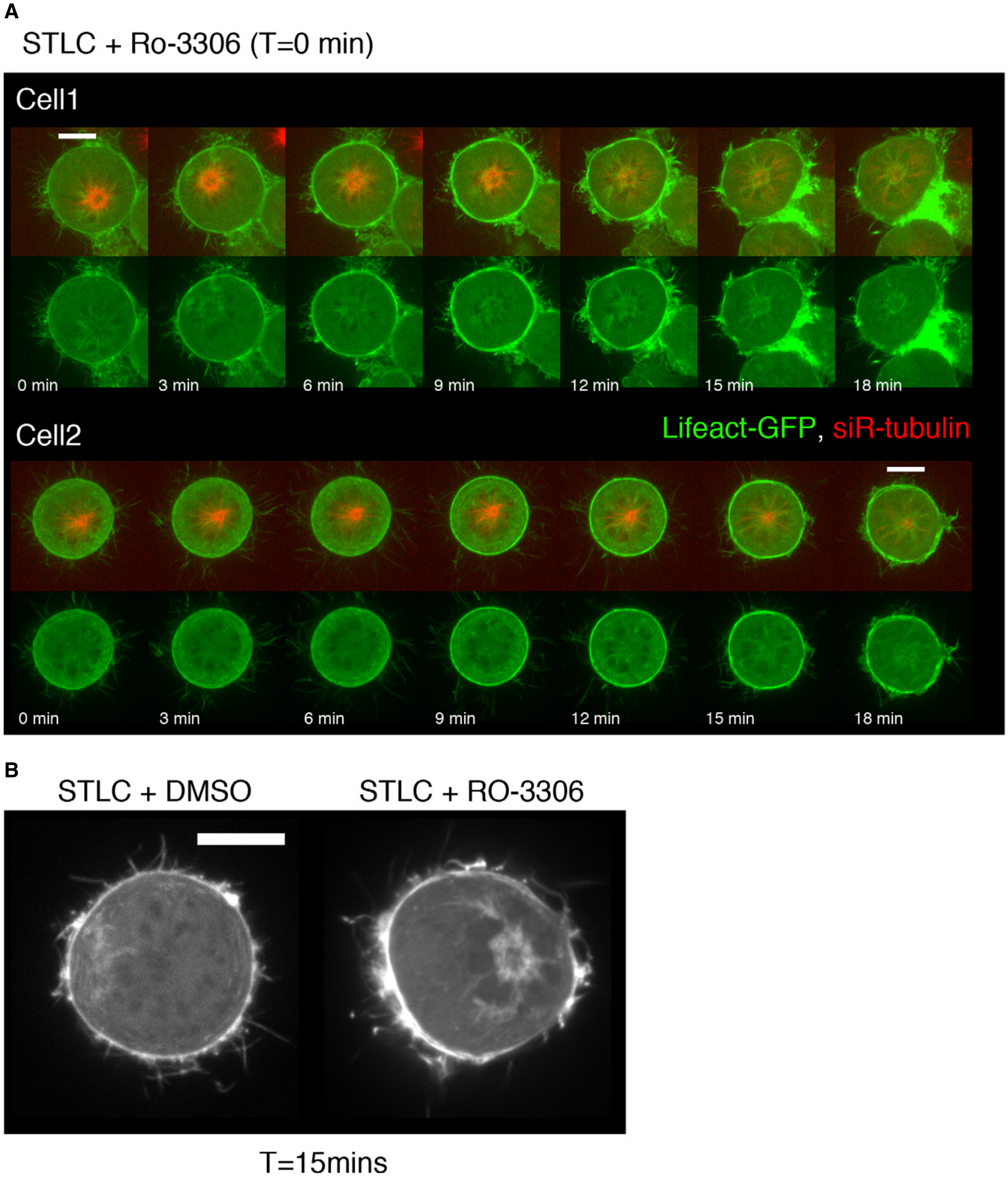

Figure EV3. Actin dynamics during forced exit in HeLa cells.

A Stills from time lapse of HeLa cells expressing Lifeact-GFP and labelled with siR-tubulin during monopolar exit, imaged every 1.5 min, showing actin accumulation around centrosomes.

B High-resolution image of Lifeact-GFP localisation in cells arrested in prometaphase (+DMSO) or cell forced to exit with Cdk1 inhibitor, for 15 min, showing actin accumulation around the centrosomes.

 © 2019 The Authors

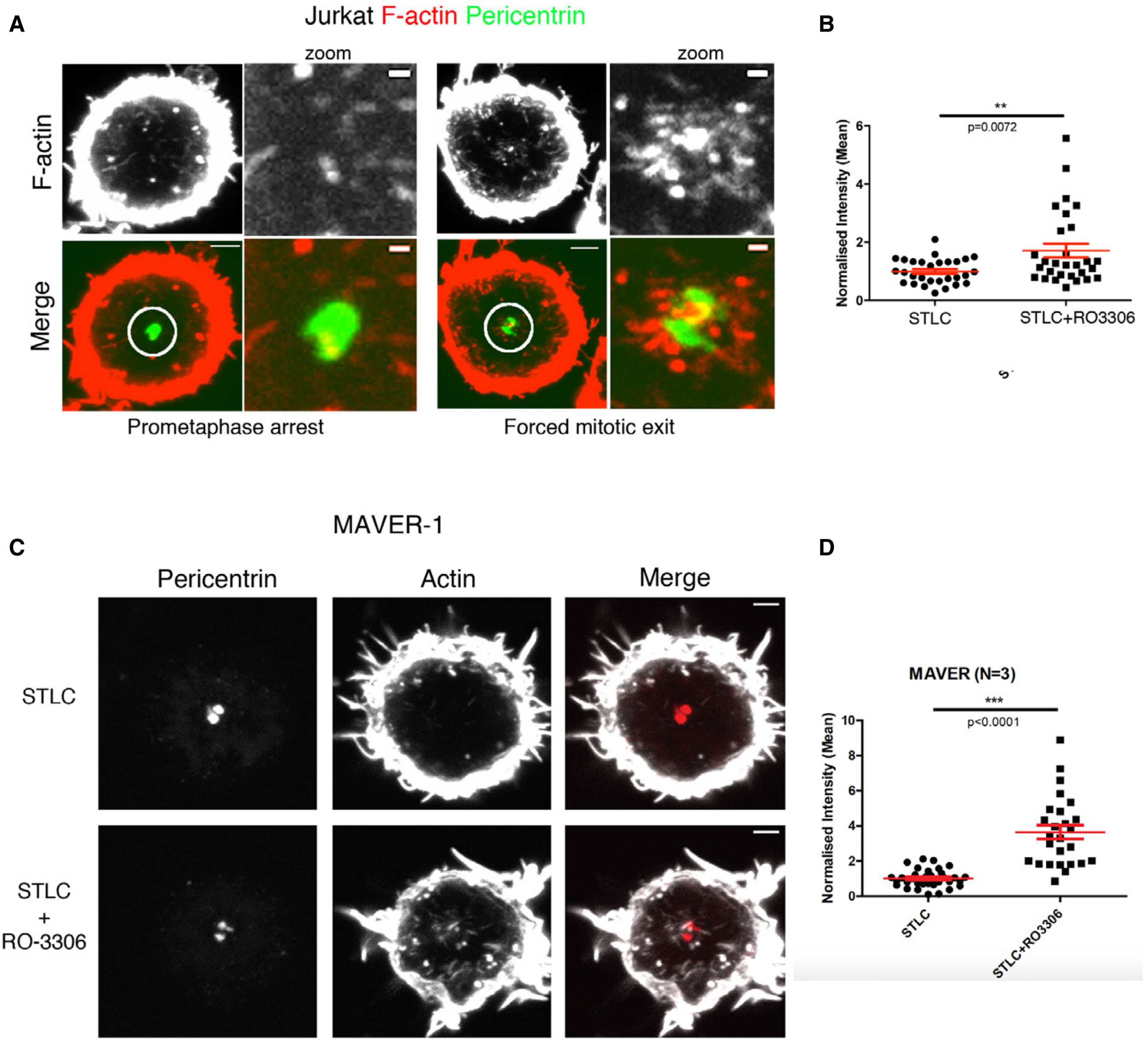

Figure EV4. Actin dynamics during forced exit in Jurkat and MAVER1 cell lines.

A Maximum projection view of Jurkat cells immunostained for F-actin (phalloidin) and pericentrin arrested at prometaphase with STLC and forced to exit mitosis with RO-3306 (10 min) showing the increase in F-actin around centrosomes during forced exit. Scale bar = 5 μm and for zoom = 1 μm.

B The level of actin around the centrosome in (A) was quantified and normalised relative to metaphase and shows an increase during forced mitotic exit. STLC arrest = 1.000 ± 0.08018, N = 29; STLC+RO-5 min = 1.713 ± 0.2361, N = 29, Welch's t-test, P = 0.0072.

C Maximum projection image of MAVER1 cells arrested in prometaphase with STLC and forced to exit with STLC+RO-3306 addition (10 min), immunostained with pericentrin (for centrosomes) and phalloidin (F-actin), showing the increase in actin around the centrosomes during the forced exit. Scale bar = 5 μm

D Quantification for MAVER1 cells stained as above, showing the increase in actin during forced mitotic exit. STLC arrested = 1.000 ± 0.08336, N = 35; STLC+RO-5 min = 3.647 ± 0.3894, N = 26; Welch's t-test, ****P < 0.0001. Error bars indicate standard error of the mean.

© 2019 The Authors

Figure EV5. Arp2/3-dependent actin nucleation around centrosomes during monopolar exit.

A Representative image of cells arrested in prometaphase with STLC incubated for 2 h with DMSO or 0.2 mM CK666 was imaged every 90 s as they were forced to exit mitosis as the result of RO-3306 addition. *n* = 26 cells (DMSO) and 27 cells (CK666) from two independent experiments. Scale bar = 5 µm and for zoom = 2 µm.

B Quantification of actin around centrosome for cells treated with DMSO or 0.2 mM CK666 prior to forced exit shows that pre-treatment with 0.2 mM CK666 leads to a failure to accumulate actin around the centrosome during exit. *n* = 26 cells (DMSO) and 27 cells (CK666) from two independent experiments. Error bars indicated standard deviation.

C Quantification of tubulin around centrosomes for cells treated with DMSO or 0.2 mM CK666 prior to forced exit shows that pre-treatment with 0.2 mM CK666 leads to a failure to decrease tubulin around the centrosome during exit. *n* = 26 cells (DMSO) and 27 cells (CK666) from two independent experiments. Error bars indicated standard deviation.

© 2019 The Authors

© 2019 The Authors

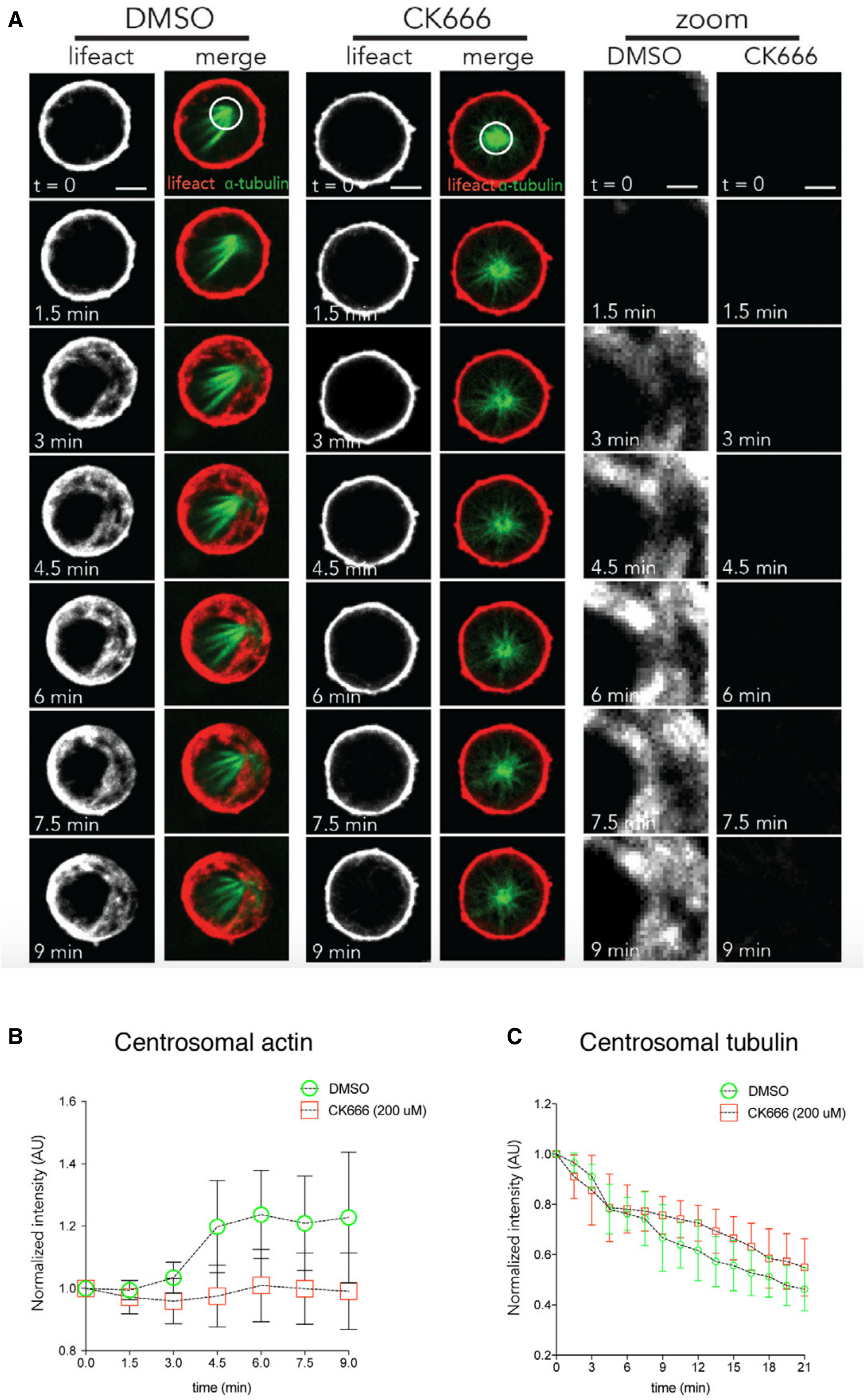
A
DMSO
CK666
zoom
lifeact
merge
lifeact
merge
DMSO
CK666
lifeact α-tubulin
t = 0
1.5 min
3 min
4.5 min
6 min
7.5 min
9 min
B
Centrosomal actin
DMSO
CK666 (200 uM)
Normalized intensity (AU)
time (min)
C
Centrosomal tubulin
DMSO
CK666 (200 uM)
Normalized intensity (AU)
time (min)

Figure EV5.

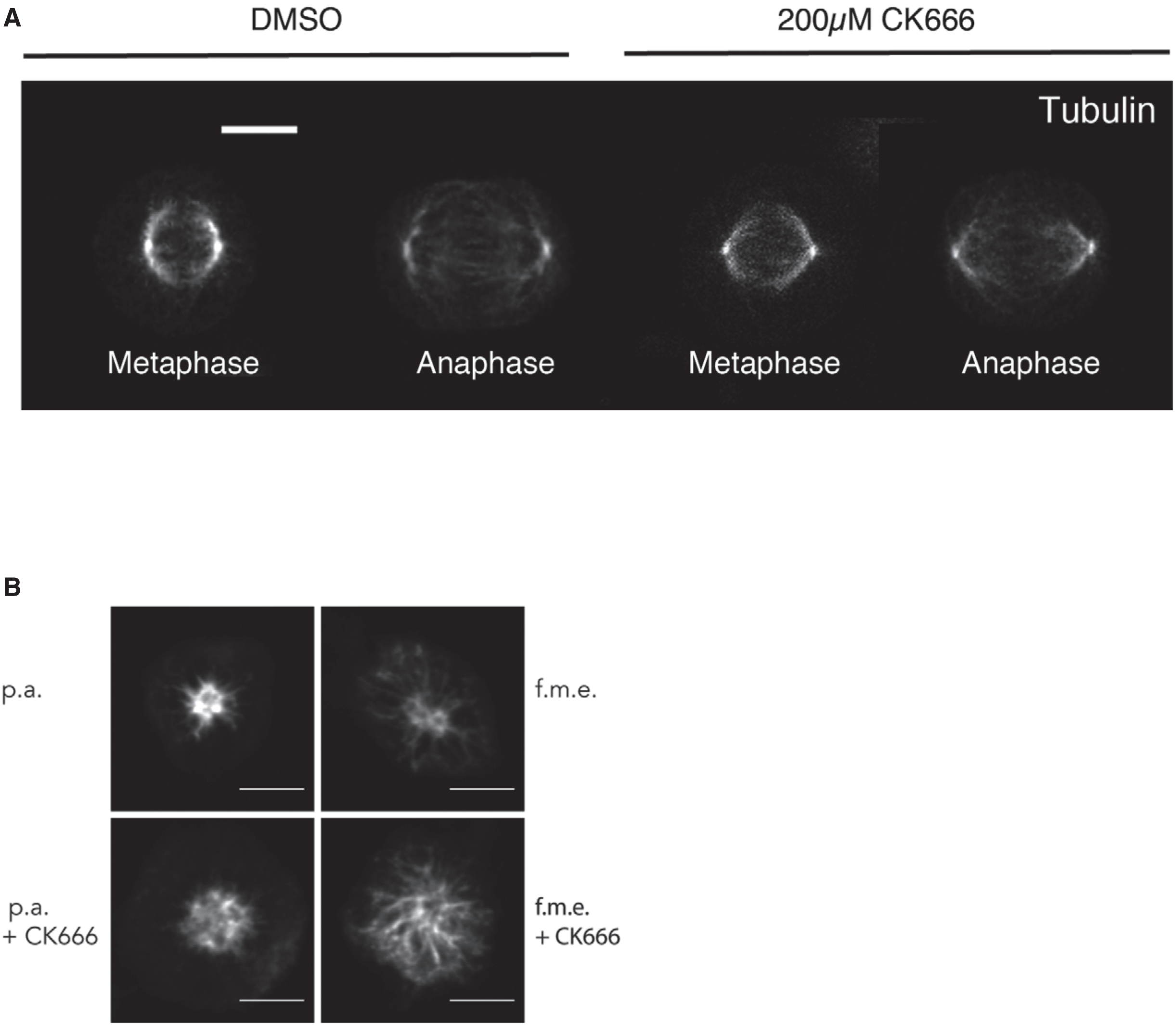

Figure EV6. Arp2/3-dependent actin nucleation around centrosomes during bipolar exit.

A Maximum projection (2 *z*-slices) view of HeLa cells pre-treated with DMSO and 0.2 mM CK666 for 15 min during their mitotic exit showing that treatment with CK666 leads to a failure to decrease tubulin during anaphase. Scale bar = 10 μm.

B Maximum projection view of HeLa cells pre-treated with DMSO and 0.2 mM CK666 during monopolar exit showing that treatment with CK666 leads to a failure to decrease tubulin during forced exit. Scale bar = 10 μm.

 © 2019 The Authors

 © 2019 The Authors

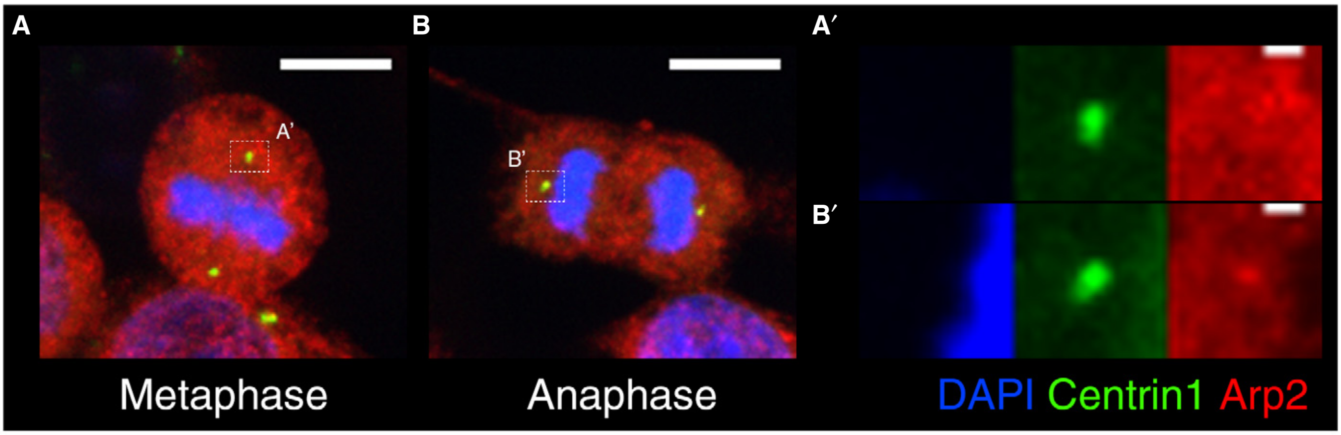

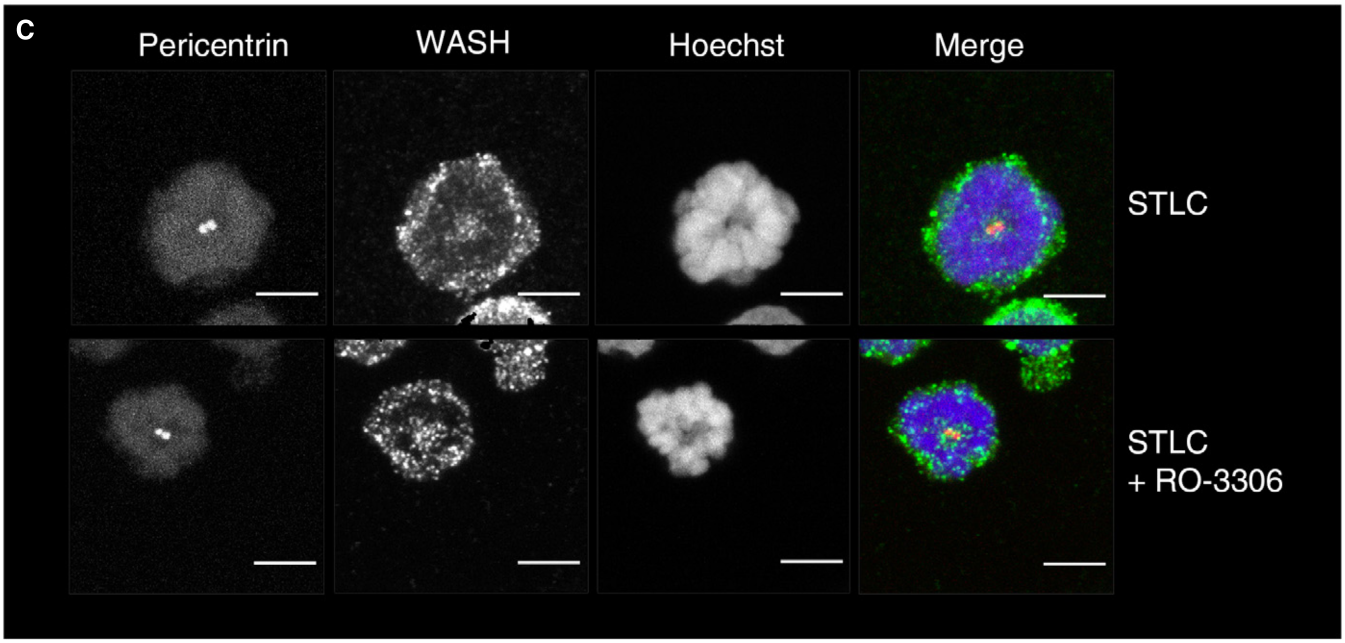

Figure EV7. Localisation of Arp2 and WASH during mitotic exit.

A, B Representative image of HeLa cell expressing centrin 1-GFP in metaphase (A,A′) and anaphase (B,B′) immunostained for Arp2, showing increased centrosomal Arp2 localisation at anaphase. Scale bar = 10 μm, zoom = 1 μm.

C Maximum projection view of MAVER1 cells immunostained with WASH1 along with pericentrin (for centrosomes) and Hoechst, in STLC-treated prometaphase-arrested cells and STLC+RO-3306-treated cells, showing the localisation of WASH around centrosomes during these stages. Scale bar = 5 μm.

© 2019 The Authors
