# Supplementary material for: Local actin nucleation tunes centrosomal microtubule nucleation during passage through mitosis
Source: EMBO J. 2019 Apr 23;38(11):e99843. doi: 10.15252/embj.201899843 (PMC6545563; doi:10.15252/embj.201899843)
Supplement: Supplementary file 3 — Movie EV2 [file EMBJ-38-e99843-s003.zip › Movie_EV2.docx]

Movie EV2:

Representative time-lapse of another Hela cell expressing Lifeact-GFP imaged every 30 seconds. Arrows point to actin in the presumptive centrosomal region. T=0 is one frame before anaphase onset. Time in seconds, Scale bar – 10µm.
