# Supplementary material for: Local actin nucleation tunes centrosomal microtubule nucleation during passage through mitosis
Source: EMBO J. 2019 Apr 23;38(11):e99843. doi: 10.15252/embj.201899843 (PMC6545563; doi:10.15252/embj.201899843)
Supplement: Supplementary file 4 — Movie EV3 [file EMBJ-38-e99843-s004.zip › Movie_EV3.docx]

Movie EV3:

Representative time-lapse of Hela cells imaged with siR-actin, every 1 min. Arrows point to actin in the presumptive centrosomal region. Note that with siR-actin, this pool of actin around centrosomes persists for a longer time. T=0 is one frame before anaphase onset. Time in minutes, Scale bar – 10µm.
