# Supplementary material for: Local actin nucleation tunes centrosomal microtubule nucleation during passage through mitosis
Source: EMBO J. 2019 Apr 23;38(11):e99843. doi: 10.15252/embj.201899843 (PMC6545563; doi:10.15252/embj.201899843)
Supplement: Supplementary file 5 — Movie EV4 [file EMBJ-38-e99843-s005.zip › Movie_EV4.docx]

Movie EV4:

Representative time-lapse of a Hela cell arrested in prometaphase with STLC, expressing Lifeact-GFP (green) and stained with siR-tubulin (red) imaged every 90 seconds following RO-3306 addition at t=-1min. Arrows point to actin accumulation around the centrosomal region. Time in minutes, Scale bar – 10µm
